# Nanoparticle Formulations of Antioxidants for the Management of Oxidative Stress in Stroke: A Review

**DOI:** 10.3390/biomedicines11113010

**Published:** 2023-11-09

**Authors:** Sara Salatin, Mehdi Farhoudi, Afsaneh Farjami, Solmaz Maleki Dizaj, Simin Sharifi, Shahriar Shahi

**Affiliations:** 1 Neurosciences Research Center (NSRC), Tabriz University of Medical Sciences, Tabriz 51666-53431, Iranfarhoudi_m@yahoo.com (M.F.); 2 Pharmaceutical and Food Control Department, Faculty of Pharmacy, Tabriz University of Medical Sciences, Tabriz 51666-53431, Iran; 3 Dental and Periodontal Research Center, Tabriz University of Medical Sciences, Tabriz 51666-53431, Iran

**Keywords:** stroke, brain, blood–brain barrier (BBB), oxidative stress, reactive oxygen species (ROS), nanoparticles

## Abstract

Stroke is currently one of the primary causes of morbidity and mortality worldwide. Unfortunately, there has been a lack of effective stroke treatment. Therefore, novel treatment strategies are needed to decrease stroke-induced morbidity and promote the patient’s quality of life. Reactive oxygen species (ROS) have been recognized as one of the major causes of brain injury after ischemic stroke. Antioxidant therapy seems to be an effective treatment in the management of oxidative stress relevant to inflammatory disorders like stroke. However, the in vivo efficacy of traditional anti-oxidative substances is greatly limited due to their non-specific distribution and poor localization in the disease region. In recent years, antioxidant nanoparticles (NPs) have demonstrated a clinical breakthrough for stroke treatment. Some NPs have intrinsic antioxidant properties and act as antioxidants to scavenge ROS. Moreover, NPs provide protection to the antioxidant agents/enzymes while effectively delivering them into unreachable areas like the brain. Because of their nanoscale dimensions, NPs are able to efficiently pass through the BBB, and easily reach the damaged site. Here, we discuss the challenges, recent advances, and perspectives of antioxidant NPs in stroke treatment.

## 1. Introduction

To date, the global burden of stroke-related disability and death has continued to increase [1,2]. There are a wide variety of risk factors for stroke such as genetic predisposition, old age, smoking, severe obesity, unhealthy diet, and stress. Excitotoxicity, oxidative stress, inflammation, and apoptosis have been identified as key contributory factors influencing lesion progression [3,4]. Reactive oxygen species (ROS) are known to be essential in regulating various physiological functions. However, events associated with spontaneous or pharmacological reperfusion result in an imbalance between ROS production and elimination, leading to a physiologic process termed oxidative stress. In past years, various therapeutic strategies have been used to disrupt ROS formation or promote ROS scavenging activity. Antioxidant therapy has been suggested to attenuate the extent of ischemic brain injury with varying degrees of success [5,6]. An antioxidant agent inhibits the oxidation of suitable substrate and acts against the harmful effects of free radicals. Apart from their endogenous origin, antioxidants are compounds that detoxify free radicals by catalytic mechanism and are significantly more effective than nonenzymatic antioxidants at lower doses. However, antioxidants have received very limited success until now, because of their poor water solubility, low permeability, and instability in the gastrointestinal tract. Therefore, there is no effective antioxidant therapy for stroke, partly due to the challenges in delivering antioxidant agents to the impaired brain sites.

Advances in nanotechnology shed light on the growing therapeutic potential of nanoparticles (NPs) in stroke treatment. A combination of existing antioxidants with NPs can be a solution to address these challenges [7,8]. Antioxidant NPs have multiple benefits including stable anti-oxidative effect, good pharmacokinetics, and intrinsic ROS-scavenging capabilities. There is great demand in developing NPs with antioxidant properties for stroke therapy. This review mainly highlights the mechanisms by which NPs have improved antioxidant therapies for stroke management.

## 2. Oxidative Stress in Stroke

Stroke is an acute disease characterized by a sudden interruption of the blood supply to the brain tissue, resulting in long-lasting alterations in the neuronal circuitry and functional abilities [6,9]. 

**Figure 1:**
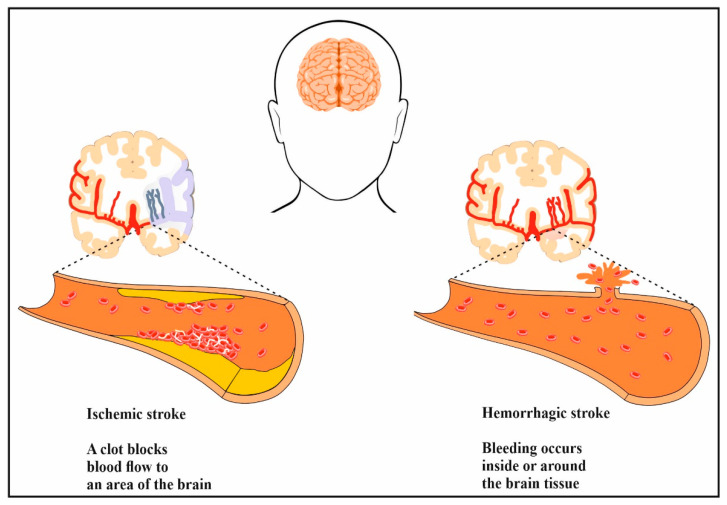
A schematic representation of the main events causing ischemic stroke, and hemorrhagic stroke.

As shown in Figure 1, stroke is categorized as either ischemic stroke or hemorrhagic stroke [10]. Ischemic stroke or cerebral ischemia is a condition that occurs when there is a blockage in the blood flow to an area of the brain. Pro-inflammatory species play an important role in the pathogenesis of ischemic stroke. A hemorrhagic stroke occurs when a blood vessel supplying the brain leaks or ruptures, causing bleeding into and around the brain [11,12]. After a stroke, millions of neural cells are lost and injured cells cannot be completely repaired or replaced by endogenous cells. Stroke still carries high morbidity and mortality rates despite significant advances in stroke therapy. Reperfusion therapy with recombinant tissue plasminogen activator (rt-PA) remains the cornerstone of acute ischemic stroke management [13,14]. However, there is an urgent need for scientists to develop novel treatment options for the great majority of stroke patients who are not eligible to receive rt-PA.

The main hurdle for developing brain drug delivery is the inability to overcome the blood–brain barrier (BBB) [15]. The BBB is a highly specialized barrier between the brain and peripheral blood circulation, protecting the neural tissue from toxins and pathogens. The BBB is composed of astrocytes, pericytes, and vascular endothelial cells, regulating the movement of substances and therapeutic agents from blood circulation into the central nervous system (CNS) [16]. Although the structural integrity of the BBB is compromised in later phases of stroke however, it still limits the penetration of therapeutic molecules for effective stroke therapy. The drugs should be given in very high doses that may, in turn, put patients at increased risk for adverse effects. Because of the time dependent nature of treatment, site-specific delivery of drugs is the most important parameter after ischemia [1]. 

**Figure 2:**
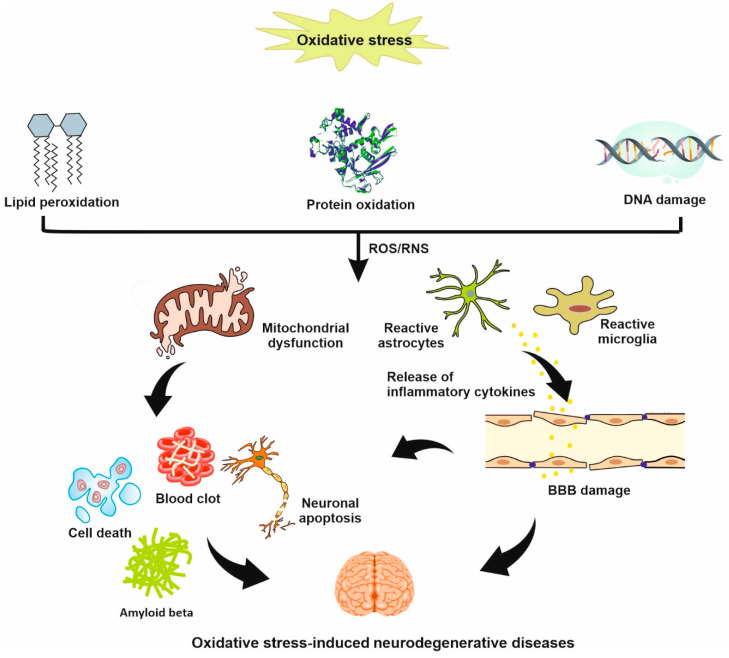
A schematic representation of the effect of oxidative stress in the development of neurodegenerative diseases/injuries such as AD, PD, and stroke.

Oxidative stress remains a central component of several neurodegenerative diseases/injuries such as Alzheimer’s disease (AD), Parkinson’s disease (PD), and stroke (Figure 2). Recently, the role of oxidative stress in brain damage due to stroke has been an important topic in stroke research. In the normal tissue, free radicals are generated as a normal product of cellular metabolism, gene transcription, and cell signaling but a balance between production and removal of ROS is provided by the endogenous antioxidant defense system [17]. Increased level of ROS has been found to be associated with obvious oxidative stress and alterations in the structure of DNA, proteins, and lipids (Figure 2). In general, reactive species found in biological organisms are called RONS and are classified into three classes: nitrogen-centered (RNS), oxygen-centered (ROS), and non-radical species that are either oxidizing agents or easily converted into radicals [18]. The major sources of ROS/RNS production are mitochondria, lysosomes, peroxisomes, endoplasmic reticulum, and plasma membrane. Free radicals are very unstable species and react easily with other molecules. Currently, lipid peroxidation is considered as the main mechanism of oxidative damage by ROS, leading to cell death. Lipid peroxides are unstable lipid radicals, which are derived from the oxidation of polyunsaturated fatty acids and can be converted to a different composition such as malondialdehyde (MDA). MDAs are highly cytotoxic and can cause irreversible damage to the membrane-bound receptors, enzymes, and transfer mechanisms [19].

The brain is highly susceptible to ischemic damage and associated oxidative stress, which is explained by its high metabolic activity, high level of readily oxidized fatty acids, and relatively low antioxidant defenses [20]. The major target of oxidative stress is the cerebral blood flow, as it plays a central role in the pathogenesis of ischemic brain injury after a cerebrovascular attack. Cerebral ischemic injury leads to increased ROS production, redox system disequilibrium, inactivation of detoxification mechanisms, and overproduction of oxidants. It has been shown that a high level of MDA is associated with poor functional recovery in patients with acute ischemic stroke [21,22]. In stroke, oxidative stress results in mitochondrial dysfunction, neuroinflammation, and glutamate excitotoxicity, leading to cell apoptosis/necrosis, BBB breakdown, autophagy, and edema formation [23]. Dysfunctional mitochondria is believed to be the main source of ROS production and disrupts energy needed for essential functions in cells, causing cell death [24]. Microglia and astrocytes are quickly activated following stroke, leading to the production of high levels of inflammatory cytokines (Figure 2) [25]. In recent years, antioxidant therapy for stroke has made few advances. More research is needed to determine the source of free radicals as well as cessation of oxidative stress in stroke. During the last decades, the effect of antioxidant NPs to improve stroke therapy has been extensively examined. In light of these findings, we highlight the advantages and disadvantages of antioxidant NPs in reducing neurological injuries after stroke.

## 3. Antioxidant NPs in Stroke

The endogenous antioxidant system is a specific defense mechanism that protects the body from the harmful effect of free radicals. In the mammalian body, the endogenous antioxidant defense system is capable of neutralizing the toxic effects of oxidative stress by both enzymatic (such as superoxide dismutase (SOD), catalase (CAT), glutathione peroxidase (GSH-Px) and glutathione-S-transferase (GST)) and non-enzymatic (such as glutathione) mechanisms [26,27]. Among these, the first line of defense is the CAT that decomposes hydrogen peroxide (H_2_O_2_) before it forms free radicals. Moreover, external antioxidant sources can protect our cells from oxidative damage mainly by targeting cellular signaling pathway, resulting in a subsequent up-regulation of the endogenous antioxidant defense system [28]. In this regard, free radicals have attracted considerable interest in recent time in assessing the therapeutic effects of antioxidants. The effects of antioxidants can be described as an antioxidant enzyme-mimetic mechanism. Antioxidant delay or inhibit cellular damage mainly by (i) inhibiting ROS generation; (ii) scavenging ROS; or (iii) increasing ROS degradation [28,29]. 

Antioxidant therapy has also been suggested as an excellent candidate for stroke treatment. Effective antioxidant therapy can be based on the upregulation of endogenous antioxidants or on the delivery of exogenous antioxidants. Many natural and synthetic antioxidants have been studied in detail for the treatment of various neurological diseases caused by oxidative stress. The most frequently examined natural antioxidants include flavonoids, curcumin, vitamin E, vitamin C, glutathione, and β-carotene, while commonly used synthetic antioxidants are lipoic acid, Edaravone, Tempol, and N-acetylcysteine [30]. However, the therapeutic potential of antioxidants is limited by their short half-lives, low penetration through the BBB, and poor site-specific brain targeting, thereby leading to their limited bioavailability [31]. Therefore, novel antioxidant-based strategies need to improve the therapeutic effect against oxidative stress damage, while limiting side effects.

**Figure 3:**
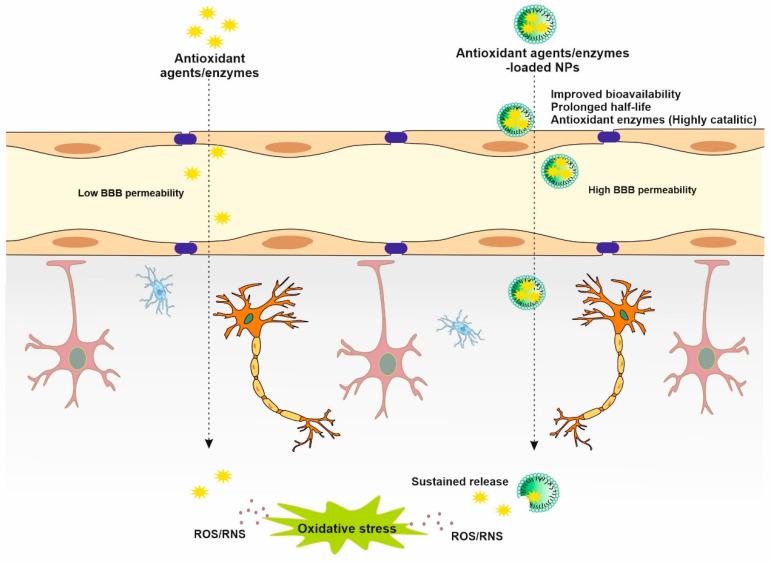
The advantages of antioxidant-based nanotherapy to improve the bioavailability of antioxidants/antioxidant enzymes in stroke treatment.

In recent decades, NPs have been emerging as an alternative antioxidant strategy in the treatment of stroke [31]. NPs are colloidal particles with a size range of 1–100 nm which can be used as delivery vehicles for therapeutic agents. NPs substantially show distinct and unique properties compared to their bulk counterparts due to quantum effects and larger surface area-to-volume ratio [32,33]. There are two general methods for the synthesis of NPs: (1) top-down and (2) bottom-up methods. In the top-down approach, bulk materials are decomposed into smaller units and then converted into NPs. Examples of this approach are grinding/milling, chemical vapor deposition, and physical vapor deposition. The bottom-up approach deals with the build-up of NPs from relatively simpler substances using different techniques such as sol gel, green synthesis, spinning, and biochemical synthesis. The reactivity, toughness, and optical characteristics of NPs are greatly dependent on their size, shape, and structure [34]. Depending on their physicochemical characteristics, NPs are broadly divided into several categories including lipid, polymer, polysaccharide, carbon, metal, and ceramic NPs [35]. Surface modification with poly(ethylene glycol) (PEGylation) is an effective strategy to increase the hydrophilicity and stability of NPs [36]. In recent years, significant efforts have been made to improve the stability, half-lives, and sustained effect of natural or synthetic antioxidant agents/enzymes in the target tissue by exploring nanocarriers of different materials, conjugates, and complexes. NPs capable of protecting antioxidant agents/enzymes from hydrolysis, achieving the targeted delivery and controlled release of cargoes at the desired site, and improving bioavailability [37]. Moreover, NPs loaded with antioxidants or antioxidant enzymes where NPs act as the carrier were shown to be efficient in enhancing the antioxidant effect and providing targeted delivery of certain antioxidants that exhibit poor permeation across the BBB (Figure 3) [38]. 

Due to the inherent structural characteristics of NPs, like ultra-small size and high surface-to-volume ratio, they have higher number of active electrons on the outer surface, leading to increased catalytic activity (e.g., antioxidant activity) [39]. NPs of different composition such as metal-based (i.e., gold, platinum) NPs, organic (i.e., melanin, lignin), and metal oxides (i.e., cerium oxide) show intrinsic redox activity which is sometimes connected with trapping of radicals and/or with SOD- and CAT-like activities [40]. Moreover, potent antioxidants can be obtained by anchoring small-molecule antioxidants on redox inactive NPs. A wide variety of biological agents can be loaded into NPs for antioxidative treatment such as small-molecule ROS scavengers, SOD, and CAT [41]. Antioxidant NPs can be classified as ROS scavenger NPs, and as vehicles to carry free radical scavengers, antioxidant genes, and antioxidant enzymes. NPs can mimic the activity of intrinsic antioxidant enzymes, such as SOD-mimetic activity, suppress apoptosis, and improve cell viability after Ischemia-Reperfusion (I/R) injury [42]. More importantly, ischemic injury to the brain leads to the increased production of ROS and H^+^ concentrations, therefore pH/redox-responsive NPs are often applied to increase drug accumulation in the ischemic brain and decrease the risk of side effects [3]. Herein, we exhibit a classification of antioxidant NPs used in the management of stroke.

### 3.1. Lipid-Based NPs 

Lipid-based NPs including liposomes, solid lipid NPs (SLNs), and nanostructured lipid carriers (NLCs) contain lipid moieties and have shown considerable potential for improving the brain bioavailability of pharmaceuticals. Surfactants or emulsifiers are commonly used to stabilize lipid NPs in the dispersion media [43,44]. Liposomes are biocompatible and biodegradable spherical vesicles composed of an aqueous core surrounded by a lipid bilayer. Liposome encapsulation could increase the brain delivery of drugs through intragastric administration, providing an increased permeability across the BBB. Liposomes can carry low and high molecular weight biomolecules and improve their efficacy [45]. While hydrophilic compounds can be loaded in the aqueous core, hydrophobic molecules can be inserted into the lipid bilayer [46]. In a recent study, ifenprodil liposomes have been evaluated as means of efficiently scavenging superoxide anion produced around the ischemic brain in rat model of middle cerebral artery occlusion (MCAO). This liposomal formulation could become a therapeutic strategy for stroke via efficient release of the encapsulated ifenprodil in vitro under a weakly acidic pH, which is a specific condition after cerebral I/R injury [47]. Iron overload is known as a major source of oxidative stress in the ischemic brain and contributes significantly to neural injury. Zhao et al. [48] demonstrated that lycopene nanoliposomes suppresses iron-overload and prevent oxidative-induced ischemic brain damage more efficiently than naked lycopene. Treatment with ascorbic acid- and α-tocopherol-loaded liposomes have been suggested in vivo to protect the cerebral tissues from oxidative free radical attack [49]. The utility of liposomes as a drug vehicle was also demonstrated using a MCAO rat model. Liposomes encapsulating the antioxidant baicalin have been suggested as means of efficiently scavenging ROS generated around the cerebral I/R region [50]. It was shown that borneol can improve BBB permeability and increase the content of drugs in the brain. In this regard, borneol-baicalin-liposomes reduced ischemia-induced neuronal injury by improving blood circulation and the half-life of baicalin [51]. The intravenous injection of liposome-entrapped CuZn-SOD increased SOD activities in the ischemic hemisphere and contralateral cortex of rats. A study found that liposomes, polybutylcyanoacrylate (PBCA), or poly (lactic co-glycolic acid) (PLGA) NPs loaded with SOD can protect primary neurons in vitro from oxygen-glucose deprivation (OGD) and limit reduce oxidant-mediated cell apoptosis. These NPs were directed to the CA regions of the hippocampus by functionalization with selective antibodies targeting the N-methyl-D-aspartate receptor [52]. NLCs are a new generation of lipid NPs composed of a blend of solid and liquid lipids. NLCs containing resveratrol are another example of an antioxidant formulation that have previously been demonstrated. Resveratrol NLCs have been shown to exert neuro-protective effects by ameliorating oxidative stress markers and attenuating activities of antioxidant enzymes and Na^+^ K^+^ ATPase in a MCAO rat model. Encapsulation of resveratrol in NLCs allowed for the substantial reduction in infarction, improved motor, and cognitive function compared to the saline control [53].

### 3.2. Polymer-Based NPs

Polymer-based NPs are colloidal particles composed of natural or synthetic polymers such as PLGA, polylactide (PLA), poly(amidoamine) (PAMAM), or poly(methyl methacrylate) (PMMA) are widely used synthetic polymers in NP preparation [54]. The particular characteristics of polymeric NPs include the feasibility of scale-up, high stability in biological fluids, the possibility to functionalize and modulate polymer degradation and the release of the loaded cargo as a function of internal/external stimuli [54]. Two major categories of polymeric NPs are recognized as nanospheres and nanocapsules. Nanospheres are matrix systems in which the drug is dispersed throughout the structure, whereas nanocapsules are vesicular systems consisting of an inner liquid core (oil or water) in which the drug is surrounded by a solid polymeric shell and nanospheres are matrix particles with solid mass [55]. In this case, even though polymeric NPs do not provide direct antioxidant activity, but numerous attempts have been performed to assess their antioxidant capacity after stroke. PLGA has attracted considerable attention as a synthetic polymer for brain drug delivery mainly due to its biocompatibility, biodegradability, and sustained drug release profile. In a recent study, PLGA NPs were reported as a novel therapeutic strategy for delivering quercetin, a natural herb origin antioxidant agent, to the brain of I/R induced young and aged rats. This nanoformulation resulted in significant down regulation of Inducible nitric oxide synthase (iNOS) and caspase-3 activities and increased neuronal count in the damaged region [56]. Tanshinone IIA (Tan IIA) can protect against ischemic stroke by providing anti-inflammatory and antioxidative effects. In a translational pig stroke model, intrathecal administration of Tan IIA-NPs resulted in antioxidative effects, prolonged survival of induced neural stem cells post-transplantation, and accelerated recovery of neurological function [57]. RVG29 peptide-modified PEG–PLGA NPs demonstrated superior targeting ability to the brain in a cerebral ischemia rat model. Intranasal administration of RVG29-modified PEG–PLGA NPs encapsulating the neuroprotective agent, baicalin, after ischemic brain injury was found to significantly alleviate oxidative stress and inhibit inflammation [58]. In another similar work, acute testing of Tan IIA-PEG-PLGA NPs mitigated free radical-induced brain damage, resulting in less severe functional deficits post-ischemic stroke [59]. The exogenous delivery of the native form of antioxidant enzymes to prevent the damaging effects of ROS has been limited because of its short half-life and poor permeability across the BBB. Different strategies have been demonstrated to address these issues. In this regard, SOD and CAT loaded in PLGA NPs were shown to attenuate mitochondrial dysfunction, neuronal cell apoptosis, and secondary injury in a rat contusion model of severe spinal cord injury. Intravenous administration of NPs reduced mitochondrial ROS activities, increased mitochondrial membrane potential, and also higher adenosine triphosphate (ATP) production capacity at the lesion site [60]. Reddy’s group investigated the antioxidant efficacy of the SOD encapsulated in sustained release biodegradable PLGA NPs in a rat transient focal cerebral I/R injury model. Intracarotid administration of nano-SOD improved survival compared to control groups (saline or SOD solution). There was evidence of regeneration and neuronal recovery with time [61]. A separate study in a thromboembolic rat stroke model that has received t-PA and nanoPLGA-SOD/CAT demonstrated the migration of progenitor cells from the subventricular zone to the rostral migratory stream, promoting neurogenesis. In contrast, this process was inhibited in animals treated with t-PA alone or untreated control. Significantly, t-PA+nano-SOD combination treatment inhibited edema formation by protecting the BBB from reperfusion injury [62]. Polymeric NP-mediated CAT delivery improved neuronal recovery from H_2_O_2_-induced oxidative stress and facilitated the recovery of neurons better than free CAT, suggesting possible applications in ischemia for ameliorating the level of irreversible brain injury [63]. Wu et al. [7] developed multifunctional NPs using a ROS-reactive PEG-terminated poly (2,2′-thiodiethylene 3,3′-thiodipropionate) (PTT) polymer and further conjugated with AMD3100, which recognizes CXCR4. NPs were able to accumulate in the ischemic brain tissue through both thrombin-triggered shrink ability and AMD3100-mediated targeted delivery. They also demonstrated that the delivery of glyburide via NPs effectively decreased mouse infarct area, and improved neurological functions. Wang et al. [64] fabricated mPEG-b-PLA NPs containing curcumin and tested their usefulness both in vitro and in vivo. The prepared NPs alleviated oxidative stress-induced microvascular endothelial cells damage. In vivo, NPs accumulated in the ischemic penumbra, reduced the infarct size, and improved function recovery. Kang and co-workers [37] developed iron-gallic acid (Fe-GA) coordination polymer NPs to achieve an impressive antioxidative and neuroprotective effect in a ischemia stroke rat model. The resulting data suggested that Fe-GA NPs decreased brain infarct volume and increased stroke recovery by scavenging reactive oxygen and nitrogen species. Li’s group [5] evaluated ROS-responsive PAMAM NPs conjugated with COG1410, a BBB-targeting peptide, and salvianic acid A, an antioxidant agent, in stroke treatment. According to their results, NPs successfully were able to penetrate the BBB, improve over two times the infarct area, and reduce the damage caused by ROS in MCAO mice. On the other hand, these NPs did not show any toxicity on other organs of the body. In another report, encapsulation of t-PA in self-assembled polyion complex NPs showed potential in extending the narrow therapeutic window of t-PA. Further, the NPs matrix was covalently conjugated with the strong antioxidant 4-amino-2,2,6,6,-tetramethylpiperidine-1-oxyl (4-amino TEMPO) moieties to suppress I/R-induced oxidative stress [65]. 

Recently, stimuli-responsive polymeric NPs have exhibited great promise for the treatment of ischemic brain due to their ability to change physicochemical characteristics in response to external stimuli such as temperature, ROS, light, pH, and ionic strength. ROS responsive polymeric NPs can undergo changes in response to ROS with variations in solubility and induced degradation, allowing the release of drug. Poly(Propylene Sulphide) (PPS) is one of the polymers suggested as ROS-responsive agents in stroke therapy. In contact with ROS, its Sulphur (II) atoms change their polarity and are turned to their polar form with excellent solubility, leading to the release of loaded cargo. ROS-scavenging PEGylated polymeric NPs composed of PPS showed remarkable antioxidant and anti-inflammatory characteristics in a mouse model of stroke. Moreover, treatment with PPS NPs significantly reduced infarct volume, neuronal loss, and improved recovery of neurological function, whilst showing negligible cytotoxicity [66]. Lee’s group [67] formulated ROS-responsive copolyoxalate NPs containing vanillyl alcohol, a plant-derived antioxidant, for the treatment of ischemic brain injury. On reaching the ischemic region, NPs directly react with overproduced H_2_O_2_ and release vanillyl alcohol. When tested on a mouse model of I/R injury, NPs were able to reduce oxidative stress and induce potent anti-apoptotic and anti-inflammatory and effects. In another research work, Yoshitomi et al. [68] developed pH-sensitive NPs containing nitroxyl radicals in their core. According to the results, NPs disintegrated in response to low pH in the ischemic region, resulting in effectively scavenging ROS.

### 3.3. Polysaccharide-Based NPs

Polysaccharides are classified into positively charged (e.g., chitosan) or negatively charged (e.g., alginate, hyaluronic acid, and pectin) materials. NPs composed of chitosan are among the most studied systems for brain antioxidant delivery. Chitosan possesses cationic character and the free amino groups allow it to interact with the negatively charged cell membrane. Chitosan NPs are known to transiently open the cellular tight junctions in a reversible and transient manner, permitting an increase in drug permeation [69]. Ding and co-workers [70] demonstrated that carboxymethyl chitosan NPs can serve as a potent delivery vehicle for Acetyl-11-Keto-β-Boswellic acid (AKBA), a potent antioxidant and anti-inflammatory agent, in cerebral ischemic therapy. AKBA NPs reach the brain tissue and exert neuroprotective effect against oxidative stress more effectively than AKBA, alone. In another study, active oligosaccharide material-derived NPs were fabricated by covalently conjugating Tempol, a radical-scavenging agent, and phenylboronic acid pinacol ester on β-cyclodextrin (termed TPCD). TPCD NPs in MCAO mice successfully reduced infarct volume and improved neurological function, largely resulting from its antioxidative, anti-inflammatory, and antiapoptotic effects [71].

### 3.4. Carbon-Based NPs

Carbon-based NPs are made solely from carbon atoms and can be divided depending on their shape and geometrical structure. Famous examples of carbon NPs are graphene, nanotubes (tube), fullerenes (spherical or ellipsoidal), carbon clusters, carbon dots, and nanodiamonds [72]. It was reported that carbon-based NPs prevent damage caused by oxidative stress and reduce cerebral infarction size by 50% [73]. Fullerenes are a series of hollow carbon molecules and have been evaluated as means of efficiently scavenging free radicals of ischemic hemispheres. Results demonstrated that fullerene NPs inhibit brain oxidative/nitrosative injury and protect the brain cells against I/R damage in a rat model of ischemic stroke [74]. In another study, TaghiMohammadi et al. [75] found that fullerenol NPs can increase the SOD activity and decreases brain infarction during cerebral I/R injury in in vivo experiments. Hexasulfobutylated C60 increased NO level and reduced lactate dehydrogenase (LDH) content in MCAO rats when administered intravenously [73]. The injection of single-walled PEG-carbon nanotubes was reported to increase the expression of antioxidant enzymes in the hippocampus of normal rats [76]. PEGylated hydrophilic carbon clusters were found to function as SOD [77,78]. Moreover, PEG-carbon clusters were capable of scavenging •OH after stroke [29]. In another research work conducted by Dharmalingam et al. [79], deferoxamine bound covalently to PEG- carbon clusters reduced mitochondrial DNA damage, and heme-derived ROS formation in the mouse brain following intracerebral hemorrhage. 

### 3.5. Metal NPs

The widespread use of metallic and metal oxide NPs in many fields has increased human exposure. Metallic NPs are highly biocompatible particles and can be easily engineered to carry a wide variety of therapeutic agents due to high negative surface charge [80]. Of note, these NPs show excellent optoelectrical characteristics due to their well-known localized surface plasmon resonance effect. Metal oxides NPs are capable of participating in biological redox reactions and mimicking a large number of enzymes such as CAT, SOD, oxidases, peroxidases, ATPases, and phosphatase [81,82]. Several in vivo studies demonstrated that after oral administration [83], intravenous injection [84], intraperitoneal injection [85], and intranasal instillation [86], metallic NPs can be detected in secondary target organs such as the brain, lung, spleen, liver, and kidneys. Some metallic and metal oxide NPs, like Pt, Se, TiO_2,_ Fe_3_O_4,_ and CeO_2_ NPs (CeNPs or nanoceria), have been shown to exert potent ROS-scavenging activities due to the presence of free electrons on their surface [87]. PtNPs are capable of scavenging superoxide anion (O2^•−^) radicals and mimic the activity of antioxidant enzymes. Treatment with PtNPs significantly reduced superoxide anion generation, the infarct volume, and matrix metalloproteinase-9 activation in transient MCAO mice [88,89]. Se is an essential trace element that regulates many bodily functions such as enzyme activity, antioxidant defense, and immune response through different pathways [90]. Among various inorganic NPs, SeNPs have been extensively used due to their excellent antioxidant activities. The antioxidant effect of Se is mainly attributed to the seleno enzymes like and glutathione peroxidase and thioredoxin reductase family with ROS scavenging activity [91]. Nanoceria exert a much higher catalytic effect than cerium bulk forms, mostly due to the elevated surface to volume ratio. Nanoceria exist in two oxidation states, cerous (Ce^3+^) or ceric (Ce^4+^) ion. Nanoceria have rich surface oxygen vacancies, allowing redox cycles between its two oxidation states [92]. In addition, oxygen vacancies on the surface of NPs are generated owing to the reversible reduction of Ce^4+^ to Ce^3+^. This allows nanoceria to exhibit their unique catalytic property, mimicking the activities of SOD, CAT, and ROS/RNS scavenging activities [93]. Additionally, nanoceria are known to possess phosphatase-like, peroxidase-like, oxidase-like, and ATPase-like mimetic activity. In a research work, zeolitic imidazolate framework-8-capped nanoceria were able to inhibit lipid peroxidation, suppress the activation of astrocytes and secretion of proinflammatory cytokines, and reduce apoptosis of neurons in the mice brain tissue, achieving satisfactory antioxidant treatment against I/R injury in ischemic stroke [79]. Nanoceria may serve as a free radical scavenger and exhibit oxygen defects in their lattice structure [94]. In vitro studies demonstrated potent antioxidative, and anti-inflammatory activities of nanoceria with high Ce^3+^ to Ce^4+^ ratio. Nanoceria significantly improved neurological outcomes and survival rate after subarachnoid hemorrhage in a rodent model [95]. 

Among the existing NPs, gold NPs (AuNPs) have attracted much research interest in antioxidant therapy. Gold is considered to be chemically inert and resistant to corrosion, providing a unique perspective for nanoscale technologies and devices. Depending on the NPs size, AuNPs can show oxidative or antioxidant effect in stroke treatment. For example, 20 nm AuNPs more effectively reduce the area of cerebral infarction, while 5 nm AuNPs lead to DNA damage and enlarged infarction [96]. It was reported that 20 nm AuNPs can protect primary cortical neurons against oxygen-glucose deprivation/reperfusion injury by alleviating oxidative stress and neuronal apoptosis, while opposite results were reported for 5 nm AuNPs [97]. Co-doped Fe_3_O_4_ nanozyme can function as a potent platform blocking excessive generation or removing RONS in vitro and in vivo. Therapeutically, Co-Fe_3_O_4_ nanozyme significantly decreased infarct volume in stroke models after intravenous administration [98].

### 3.6. Ceramic NPs

Ceramic NPs are nonmetallic solids with amorphous, polycrystalline, dense, porous or hollow forms. These NPs are basically comprised inorganic compounds such as hydroxyapatite, zirconia, silica (SiO_2_), titanium oxide (TiO_2_), and alumina (Al_2_O_3_) and can be synthesized via heat and successive cooling. An important category of ceramic NPs are silica-based NPs that can be categorized as nonporous (solid) or mesoporous NPs. Thymoquinone has been proved to possess antioxidant and anti-inflammatory effects. The encapsulation of thymoquinone in mesoporous silica nanocarriers increased the activities of SOD, CAT, and glutathione content, while decreased the malondialdehyde content in the brain of rats, proving its antioxidant effect [99].

### 3.7. Other NPs

Flavonoids have long been reported as a class of secondary plant phenolics with potent antioxidant effects. Nanocurcumin has been reported to be used clinically in the treatment of neurological disorders, such as amyotrophic lateral sclerosis [100] and multiple sclerosis [101], showing antioxidant effects. Of note, studies have shown considerable safety and tolerability of nanocurcumin in human subjects. Nanocurcumin can offer a basis for our perspective on the future of stroke treatment. In a research conducted by Saghari et al. [102], nanobetanin and nanocurcumin reduced ROS content and improved the activity of antioxidant enzymes (e.g., SOD) in cerebral I/R rats after oral administration. Melanin is recognized to function as a potential radical scavenger and it is believable that melanin NPs can be studied clinically in the treatment of RONS-associated diseases in the future. Liu et al. [103] found that bioinspired PEG-coated melanin NPs can suppress the expression of inflammatory mediators and protect the ischemic brain from RONS-induced injury in both in vivo and in vitro experiments. Another in vivo work supports the evidence that betulinic acid (BA) NPs improve the delivery of glyburide to the brain, leading to antioxidative benefits significantly higher than glyburide and BA NPs in stroke-bearing mice [3]. Similarly, AMD3100-conjugated BA were assessed as a therapeutic platform to enable NA1 release in the acidic ischemic tissue through chemically converting BA to betulinic amine and targeted drug delivery through interaction with CXCR4 [1]. Coating the surface of NPs with leukocyte membranes could become a promising strategy to overcome biological barriers and improve NP-mediated drug delivery for the treatment of ischemic stroke. Feng et al. [104] prepared mesoporous Prussian blue nanozyme coated with neutrophil-isolated cell membranes. Nanozymes demonstrated the specific targeting abilities to pass through the inflamed BBB, reach the brain parenchyma around the I/R region, and exert antioxidative effects.

## 4. Safety Concern of NPs

Although NPs claim potential use in stroke therapy, there are certain challenges in their clinical translation. At the same time, NPs have been shown to produce toxic effects. The physicochemical characteristics of NPs (e.g., size, shape, surface charge, and surface chemistry) determine their cytotoxic effects. The cytotoxic effects of NPs limit the clinical use of these systems, and it is necessary to minimize cytotoxic effects by continuously changing the physicochemical characteristics of NPs. The smaller the NPs, the larger the surface area to volume ratio, allowing their interaction with the cellular components. AuNPs with the size of 5 nm have been shown to cause oxidative stress by accumulation of NPs into the nucleus and organelles, promoting DNA damage [97]. NPs with a size greater than 100 nm are rapidly cleared by the phagocytic cells and accumulated in the liver and the spleen. In the blood stream, NPs adsorb plasma proteins on their surfaces, forming protein corona that act as signals to phagocytic receptors on immune cells, and induce phagocytosis. Several studies have reported that the nonionic, hydrophobic coating of NPs surface promotes protein adsorption. Therefore, the surface coating of NPs with hydrophilic polymers, like PEG, offers a solution to minimize immunogenicity, reduces clearance by the immune system, and hinders interactions with nonspecific organs. Hydrophobic NPs have been shown to absorb into the lipid bilayer more easily, so they exert relatively high toxic effects. The cellular membrane is negatively charged. Therefore, positively charged NPs exhibit higher cellular uptake than negatively charged NPs. The cytotoxic effects of NPs may be related to inflammatory reactions and ROS generation. For example, nanoceria exert a cytotoxic effect by producing proinflammatory cytokines [105]. Therefore, there is an urgent need to assess the cytotoxicity effect of every type of NPs.

## 5. Conclusions

NPs have shown superior therapeutic effect compared to traditional treatments. It has also been reported that the redox property of antioxidant NPs can be safely used for various therapeutic applications. Moreover, NP-mediated antioxidant delivery can protect human neurons from stroke-relevant oxidative stress. In past decades, NPs have been suggested as a solution to overcome the lack of clinical translation in stroke treatment and neural regeneration. However, translation of data from the preclinical studies to the clinic is limited by many elements like lack of use of clinical-grade cell lines, suboptimal dosage, timing, and mode of administration. Recent researches have focused on controlling the composition, size, shape, and surface chemistry of NPs due to enhance the catalytic or antioxidant efficacy of NPs. Also, their potential toxicity should be taken into consideration as a safety concern. 

## Data Availability

Not applicable.
